# Use of Murine Bioassay to Resolve Ovine Transmissible Spongiform Encephalopathy Cases Showing a Bovine Spongiform Encephalopathy Molecular Profile

**DOI:** 10.1111/j.1750-3639.2011.00526.x

**Published:** 2012-05

**Authors:** Katy E Beck, Rosemary E Sallis, Richard Lockey, Christopher M Vickery, Vincent Béringue, Hubert Laude, Thomas M Holder, Leigh Thorne, Linda A Terry, Anna C Tout, Dhanushka Jayasena, Peter C Griffiths, Saira Cawthraw, Richard Ellis, Anne Balkema-Buschmann, Martin H Groschup, Marion M Simmons, John Spiropoulos

**Affiliations:** 1 Animal Health and Veterinary Laboratories AgencyAddlestone, Surrey, UK; 2 Virologie Immunologie Moléculaires, U892, Institut National de la Recherche AgronomiqueJouy-en-Josas, France; 3 Friedrich-Loeffler-Institut, Federal Research Institute for Animal HealthGreifswald-Insel Riems, Germany

**Keywords:** BSE, mouse bioassay, prion, scrapie, transmissible spongiform encephalopathy

## Abstract

Two cases of unusual transmissible spongiform encephalopathy (TSE) were diagnosed on the same farm in ARQ/ARQ PrP sheep showing attributes of both bovine spongiform encephalopathy (BSE) and scrapie. These cases, UK-1 and UK-2, were investigated further by transmissions to wild-type and ovine transgenic mice. Lesion profiles (LP) on primary isolation and subpassage, incubation period (IP) of disease, PrP^Sc^ immunohistochemical (IHC) deposition pattern and Western blot profiles were used to characterize the prions causing disease in these sheep. Results showed that both cases were compatible with scrapie. The presence of BSE was contraindicated by the following: LP on primary isolation in RIII and/or MR (modified RIII) mice; IP and LP after serial passage in wild-type mice; PrP^Sc^ deposition pattern in wild-type mice; and IP and Western blot data in transgenic mice. Furthermore, immunohistochemistry (IHC) revealed that each case generated two distinct PrP^Sc^ deposition patterns in both wild-type and transgenic mice, suggesting that two scrapie strains coexisted in the ovine hosts. Critically, these data confirmed the original differential IHC categorization that these UK-1 and UK-2 cases were not compatible with BSE.

## INTRODUCTION

Transmissible spongiform encephalopathies (TSEs) are fatal, neurodegenerative diseases affecting animals and humans; they include bovine spongiform encephalopathy (BSE), where cattle are the natural host, and scrapie, which affects both sheep and goats. A ubiquitous feature of all TSEs is the accumulation/deposition of a pathogenic isoform (PrP^Sc^) of the cellular prion protein (PrP^C^) (31) in the central nervous system (CNS) of infected individuals. Other pathological features of these diseases include spongiform change in the brain (vacuolation), neuronal loss, gliosis and amyloid plaque formation (33).

BSE is a TSE that has infected large numbers of cattle in the UK since it was first discovered in 1986 (43, 44), and the BSE agent has been linked with TSE diseases in other species (18, 46), including humans, in the form of variant Creutzfeldt–Jakob (vCJD) disease (15, 36). Experimental transmissions have shown that sheep can be infected by BSE prions, and the clinical disease they produce is indistinguishable from scrapie. BSE is also transmissible between sheep within an experimental flock (5, 6, 22). The recognition that BSE in cattle was a zoonotic disease and that, if transmitted to sheep, it could not be differentiated from scrapie by statutory methods, provoked concerns that the UK national sheep flock might act as a secondary source of a vCJD-type of disease. In 2001, a scheme was implemented to investigate the incidence of scrapie in the national flock through active, as well as passive surveillance, and this surveillance scheme was later enhanced to include a differential diagnosis of BSE and scrapie by biochemical and immunohistochemical techniques (21).

Stack *et al*(37) retrospectively monitored for BSE characteristics in TSE in sheep cases in Great Britain between 1998 and 2004. Archived samples from 1247 sheep that had been clinical scrapie suspects and subsequently found to be positive by statutory tests between January 1998 and October 2001 were retested using discriminatory immunohistochemistry (IHC) and Western blotting methods that could distinguish natural or experimental scrapie from experimental BSE in sheep (38). Additionally a further 1121 positive scrapie cases collected between November 2001 and May 2004 were prospectively screened using the same discriminatory tests. Of these, two samples generated Western blot profiles that shared similarities with those from experimental BSE in sheep and are referred to throughout this study as UK-1 and UK-2. Despite presenting a molecular profile that was similar to experimental BSE in sheep, neither sample was considered to give a BSE-like pattern on the basis of the discriminatory IHC method. Both animals had an ARQ/ARQ PrP genotype and derived from the same farm. The contradictory outcomes of these two screening tests led these cases to be referred for bioassay by the European Union transmissible spongiform encephalopathy (EU TSE) Reference Laboratory Strain Typing Expert Group (STEG) to obtain more information about the prion(s) in these sheep.

Three wild-type mouse lines have been historically used for the bioassay: RIII, C57BL and VM lines. RIII and C57BL lines share the same *Prnp* amino acid sequence (*Prnp^a^*) while VM mice (*Prnp^b^*) differ at codons 108 and 189 (42). This difference in genotype affects both the incubation period (IP) and lesion profile (LP) of a transmitted source (13). More recently, ovine and bovine transgenic mouse lines have been incorporated into the mouse bioassay, and these models have the advantage of, in some cases, reducing the IP of the development of disease (10). Crucially, the mouse bioassay is believed to permit discrimination of the BSE agent from scrapie on primary isolation (15, 16, 26). For this purpose, the RIII line is particularly informative as BSE produces a highly reproducible LP in affected mice irrespective of the source of the BSE, the tissue used for inoculation or the titer of the inoculum (25, 26), although it has been reported recently that certain classical scrapie isolates can produce similar profiles (3). The RIII mouse line is recommended by the World Organization for Animal Health (OIE) and the Community Reference Laboratory (CRL) as the model to discriminate BSE from scrapie in ovine samples with an inconclusive discriminatory diagnosis (45), and this paper reports the transmission characteristics of UK-1 and UK-2 to a panel of wild-type and transgenic mice including RIIIs.

## MATERIALS AND METHODS

### Animals and tissues

UK-1 (PG1862/02) and UK-2 (PG0175/04) were clinical scrapie suspects submitted to the Veterinary Laboratories Agency (VLA) in 2002 and 2004, respectively, that underwent a series of discriminatory tests. A detailed description of the isolates can be found elsewhere (37). Homogenates were prepared for inoculation according to tissue availability. For primary isolation into wild-type mice, a 10% (w/v) homogenate of occipital cortex (UK-1) or brain stem (UK-2) was prepared in sterile saline. For primary isolation into transgenic mice, a 10% (w/v) homogenate of parietal cortex (UK-1) or frontal cortex (UK-2) was prepared in sterile saline. For secondary passages, brain from a mouse that succumbed to TSE during first passage was used to prepare a 1% (w/v) homogenate in sterile saline.

### Mouse inoculation and sample preparation

Sources were inoculated into wild-type mice (RIII, C57BL, VM, MR and SJL/OlaHsd). MR is a modified RIII line imported from the Roslin Institute, University of Edinburgh, and SJL/OlaHsd mice were also included as previous reports indicated that this mouse line can show similar sensitivity to BSE as transgenic mice (2).

Both UK-1 and UK-2 were also inoculated into tg338 and TgshpXI ovine transgenic mouse lines expressing a valine, arginine, glutamine (VRQ) (29) or alanine, arginine, glutamine (ARQ) (28) PrP transgene respectively, on a murine PrP-ablated background. Bioassays in tg338 mice were repeated at the French National Institute for Agricultural Research (INRA), France and in TgshpXI mice at the Friedrich-Loeffler-Institut (FLI), Germany, in order to check the robustness of the bioassay. In the UK, all animal work was carried out in accordance with the Animals (Scientific Procedures) Act 1986 under UK Home Office License 70/6310 and was approved by the local ethics committee at VLA. At INRA and FLI, all animal experimentation was carried out in accordance with the European Community Council Directive 86/609/EEC. At INRA, work was approved by the INRA Jouy-en-Josas ethics committee under license number 78–109. At FLI, work was approved by the Landesveterinär- und Lebensmitteluntersuchungsamt (LVL) Mecklenburg-Vorpommern (approval number LVL M-V/310-4/7221.3-2.1-027/02).

At VLA primary inoculations were performed using 20 µL via intra-cerebral and 100 µL via intra-peritoneal routes into 15–20 mice aged from six to ten weeks per inoculum, depending on tissue availability. At INRA and FLI only intra-cerebral challenges were performed in accordance with the standard protocols in these laboratories. Subpassages were carried out at VLA only, where inoculations were performed via the intra-cerebral route. Mice were monitored for clinical signs of disease and euthanized when a specified clinical endpoint of TSE disease had been reached. Mice were also euthanized if there was significant deterioration of their health because of other factors (intercurrent deaths). Brains were removed under sterile conditions and cut along a parasagittal plane. One-third of the brain was frozen at −80°C for biochemical analysis and serial passages while two-thirds were fixed in 10% neutral buffered formalin for at least 3 days at room temperature prior to histological processing.

### Histopathological and immunohistochemical analysis

Each brain was cut into four coronal levels to reveal the medulla, rostral medulla, midbrain, thalamic and frontal cortex levels required for lesion quantification and profiling as detailed previously (26). TSE diagnosis was made based on the presence of characteristic neuropil vacuolation on hematoxylin and eosin (H&E)-stained sections, the severity of which was further semiquantified (3). Scores were assigned, on a scale of 0–5, for specific neuroanatomical gray matter areas of the brain that were then plotted to produce LP (26). Profiles should be constructed from at least five clinically and histopathologically positive mice to be considered reliable. In cases where we were not able to meet the criteria of five animals per group, we have attempted to present as much information as possible and therefore LP were also constructed to include animals that were histopathologically positive, irrespective of their clinical status. For IHC, samples were labelled with rabbit polyclonal antibody Rb486 that recognises amino acids 221–233 of the bovine prion protein (17).

### Western blot detection

#### INRA and VLA—Bio-Rad TeSeE

Tissues were extracted and analyzed by Bio-Rad TeSeE™ Western blot (Bio-Rad Laboratories Ltd., Hemel Hempstead, Hertfordshire, UK) (French—(30); UK—according to manufacturer's instructions). Briefly, 20% (w/v) tissue homogenates were treated with proteinase-K before alcohol precipitation. After centrifugation, pellets were solubilized in Laemmli buffer, and proteins separated on 12% Bis/Tris gels, electrotransferred to membrane and labeled with antiprion antibodies Sha31 (INRA & VLA), P4 (VLA, transgenic mice) or 12B2 (24) (VLA, wild-type mice; INRA, transgenic mice). Immunoreactivity was visualized using ECL Western blotting detection reagents from Amersham Biosciences (Little Chalfont, Buckinghamshire UK).

#### FLI—SAF preparation and Western blot

A 5% brain homogenate (200 µL) [in a 0.32 M Sucrose buffer containing 0.5% (w/v) deoxycholic acid sodium salt (DOC) and 0.5% (w/v) NP40 (Nonidet-P 40)] were incubated with 50 µg/mL proteinase-K for 60 minutes at 55°C to completely digest all PrP^C^. The reaction was stopped by addition of 10 mM phenylmethylsulfonylfluoride (PMSF). After incubating with 10% sodium N-lauroyl sarcosinate and 10 mM Tris-HCl, final concentrations (ambient temperature, 15 minutes), proteins were purified by centrifugation (540 000 × g, 45 minutes) over a 10% sucrose cushion (250 µL). Pellets were solubilized in Laemmli buffer, and proteins were separated on 16% Bis/Tris gels, electrotransferred to membrane and labeled with antiprion antibody L42. Immunoreactivity was visualized following incubation with chemiluminescence substrate CDP-Star (Tropix, Bedford, MA, USA).

#### Sequencing of UK-1 and UK-2 PRNP open reading frame

DNA was extracted from 100 µL ovine blood, using the Qiagen Dneasy Blood & Tissue kit (Qiagen Ltd., Crawley, West Sussex, UK). The extracted DNA was used as a template to amplify by polymerase chain reaction (PCR) a 1.1-kbp fragment of the ovine PrP gene, which included the open reading frame. Purified PCR products were cycle sequenced using six sequencing primers. After ethanol purification and preparation of the resulting sequencing products, data was generated using the Beckman-Coulter CEQ8800 Capillary Sequencer (Beckman-Coulter (UK) Ltd., High Wycombe, UK). The sequence data was analyzed using the Staden Software package (http://staden.sourceforge.net/). Polymorphisms detected by the software were confirmed and an ovine PrP open reading frame genotype derived and compared with a reference sequence (35).

## RESULTS

### IP on primary isolation in wild-type mice

The mean IP for UK-1 and UK-2 following transmission to RIII, C57BL and VM mice are presented in Table 1 and compared with mean IP for ovine and bovine BSE. Generally the IP of UK-1 and UK-2 following primary isolation in wild-type mice were longer compared with those obtained from the same mouse lines challenged with ovine or bovine BSE in agreement with previously published data (14). It has also been reported that following primary transmission of BSE, the IP in RIII mice is shorter by approximately 100 days compared with C57BL mice (14). In our laboratory, the IP difference between the two mouse lines for UK-1 and UK-2 was extended compared with the values observed for BSE (Table 1). The attack rates of UK-1 and UK-2 in wild-type mice were generally low compared with BSE transmissions (Table 1), and this is in agreement with previous studies of classical scrapie transmissions to wild-type mice (3, 16, 26).

**Table 1:** Incubation period (IP) data in wild-type mice.

					Incubation period (days)	
					
Case ID	Brain area	Mouse line	Np/N	Nc	Mean (SD)	C57BL—RIII
UK-1	Occipital cortex	RIII	7/20	4	505 (18.1)	162
		C57BL	8/20	5	667 (46.1)	
		VM	3/20	0	—	
UK-2	Obex	RIII	7/15	3	476 (27.4)	205
		C57BL	10/15	6	680 (25.3)	
		VM	9/15	3	697 (25.1)	
Ovine BSE 1	Obex	RIII	18/20	14	388 (41.1)	116
		C57BL	16/20	10	504 (112.3)	
		VM	13/20	0	—	
Ovine BSE 2	Obex	RIII	11/20	11	342 (10.8)	
Bovine BSE	Obex	RIII	8/20	8	431 (38.6)	

Np indicates the number of transmissible spongiform encephalopathy (TSE)-positive mice irrespective of their clinical status. N indicates the total number of inoculated mice. Nc indicates the subset of Np mice that also showed clinical signs of TSE; only Nc mice were used in the calculation of incubation period data. C57BL—RIII denotes the incubation period (IP) difference between C57BL and RIII for each case. SD indicates standard deviation. BSE, bovine spongiform encephalopathy.

### LP analysis on primary isolation in wild-type mice

**Figure 1:**
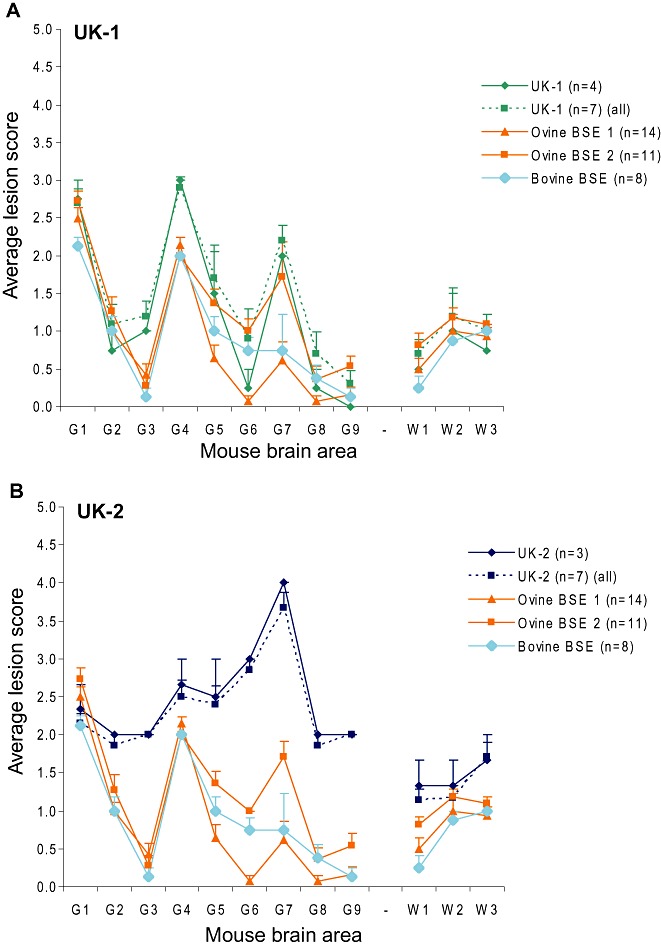
UK-1 and UK-2 LP following primary isolation in RIII mice. Profiles for RIII mice inoculated with UK-1 (**A**) and UK-2 (**B**) were obtained following quantification of specific vacuolation in nine neuroanatomical gray matter areas. Where less than five clinically and histopathologically positive mice were available, separate profiles representing all haematoxylin and eosin (H&E)-positive mice (dashed line) and mice that were both clinically and H&E positive (solid line) are plotted alongside representative bovine spongiform encephalopathy (BSE) profiles. Error bars indicate standard error of the mean. Ovine BSE 1 and Ovine BSE 2 represent duplicate bioassays using the same ovine source. The numbers of animals that contributed to each profile are given in parentheses.

The LP following transmissions to RIII mice are shown in Figure 1. The LP of UK-1 was indistinguishable from the profiles produced by BSE sources included here for comparative purposes and a large number of BSE profiles published in the literature (15, 26) (Figure 1A). The scoring area for G1 (dorsal medulla nuclei) also encompasses the cochlear nucleus, an anatomical site characteristically affected by BSE-related prions (26). Supporting Information Figure S1 shows that if the G1 scoring area is split so that the dorsal medulla nuclei (G1d) and the cochlear nucleus (G1c) are scored independently of one another, the UK-1 LP can be distinguished from ovine and bovine BSE; for BSE the score in the cochlear nucleus is equal to or higher than that in the dorsal medulla nuclei, while the opposite is observed for UK-1. This observation was not specific to UK-1. We have subsequently reanalyzed data for ten classical scrapie sources, where LP in RIII mice were previously indistinguishable from BSE based on standard LP methodology (3) and found comparable results (Supporting Information Figure S2). The LP of UK-2 in RIII mice was not consistent with ovine or bovine BSE (Figure 1B). LP following transmissions of UK-1 and UK-2 to MR mice were clearly distinguishable from BSE (Supporting Information Figure S3). These profiles are suggestive of scrapie strain ME7 in *Prnp^a^* mice (13). UK-2 was indistinguishable from BSE when isolated in SJL/OlaHsd mice; no LP was available for UK-1 as it did not transmit to this mouse line (Supporting Information Figure S3).

### Analysis of PrP^Sc^ patterns on primary isolation in wild-type mice

**Figure 2:**
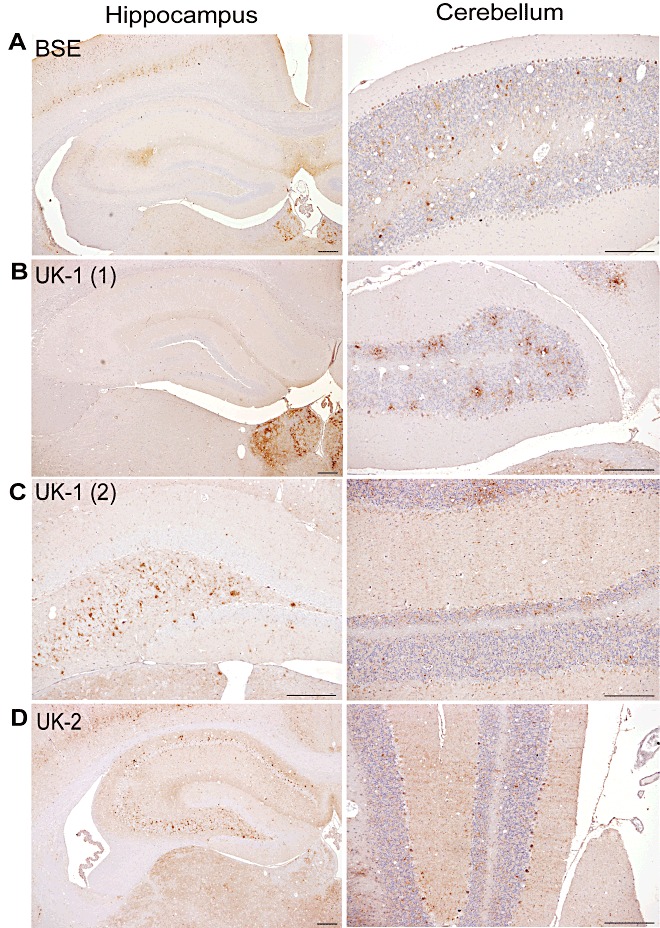
PrP^Sc^ deposition patterns of UK-1 and UK-2 following transmission to RIII mice. Representative photographs of PrP^Sc^ deposition in the hippocampus and cerebellum are shown for bovine spongiform encephalopathy (BSE) (**A**). UK-1 gave two distinct patterns termed UK-1 (1) (**B**) and UK-1 (2) (**C**). Representative PrP^Sc^ IHC labeling for UK-2 is shown in **(D**). Scale bars represent 100 µm.

IHC was performed on wild-type mice diagnosed positive by H&E analysis. Results following primary isolation in RIII mice are compared with historical ovine classical BSE transmissions to the same mouse line in Figure 2. BSE was characterized by deposition along the hippocampal fissure with a flourish of labeling at CA2 and the presence of small discrete aggregates of PrP^Sc^ in the granular layer of the cerebellum (Figure 2A). UK-1 generated two PrP^Sc^ patterns, neither of which was consistent with BSE. In one of these patterns (Pattern 1, Figure 2B) hippocampal deposition was minimal or absent while deposition in the cerebellum consisted of diffuse aggregates and granular labeling, resembling the PrP^Sc^ pattern observed for 87A (own unpub. obs.). This was designated from this point as 87A-like, as according to standard strain typing methodology in wild-type mice, stable strains cannot be defined at primary isolation. The other pattern (pattern 2, Figure 2C) was similar to scrapie strain ME7 (Supporting Information Figure S4), designated from this point as ME7-like, exhibiting deposition in the polymorph layer of the dentate gyrus and pyramidal layer in the hippocampus with fine streaks of PrP^Sc^ across the molecular layer of the cerebellum. The PrP^Sc^ pattern presented by UK-2 was also consistent with an ME7-like pattern (Figure 2D).

**Figure 3:**
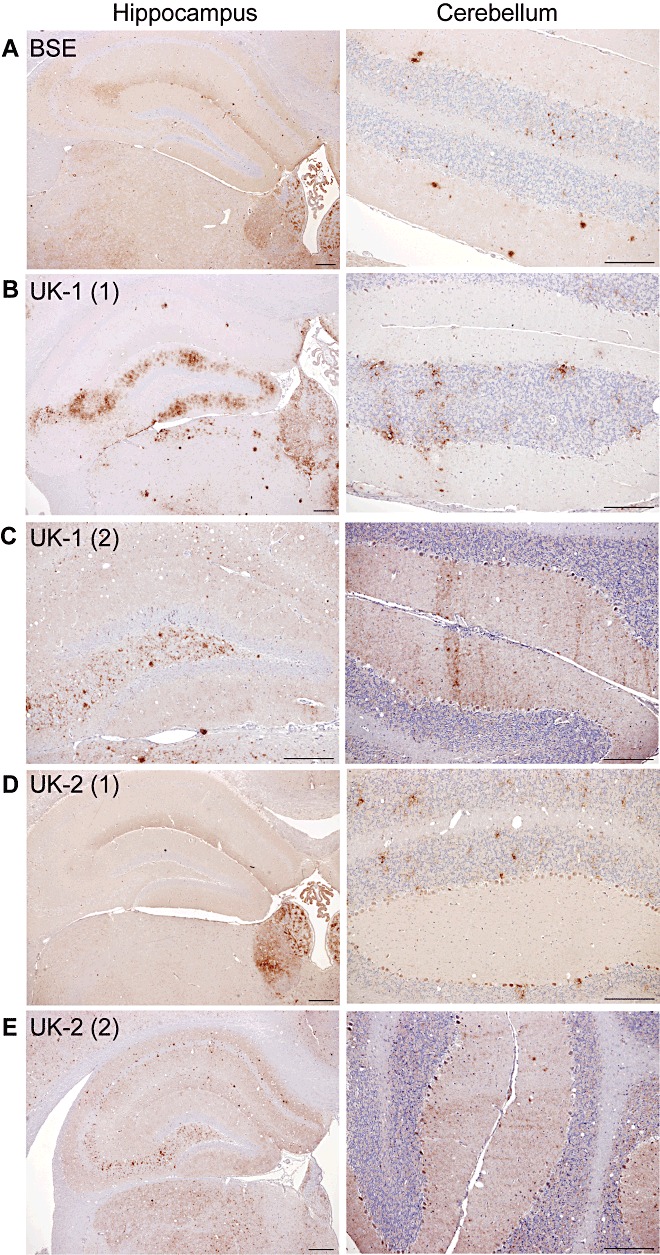
PrP^Sc^ deposition patterns of UK-1 and UK-2 following transmission to C57BL mice. Representative photographs of PrP^Sc^ deposition in the hippocampus and cerebellum are shown for BSE (**A**). UK-1 and UK-2 each gave two distinct patterns as shown, termed UK-1 (1) (**B**), UK-1 (2) (**C**), UK-2 (1) (**D**) and UK-2 (2) (**E**), respectively. Scale bars represent 100 µm.

The results of IHC analysis following primary isolation in C57BL mice are shown in Figure 3. BSE was again characterized by PrP^Sc^ deposition along the hippocampal fissure and a flourish of labeling at CA2 (Figure 3A). Small discrete aggregates of PrP^Sc^ were present throughout the granular and molecular layers of the cerebellum. In C57BL mice challenged with UK-1, two PrP^Sc^ patterns were also observed. The first was 87A-like showing deposition in the molecular layer of the dentate gyrus of the hippocampus (compare with 87A, Supporting Information Figure S4) and an absence of the small discrete aggregates in the cerebellum (Figure 3B). The second pattern was ME7-like (Figure 3C) and consistent with the pattern described for both UK-1 (Pattern 2, Figure 2C) and UK-2 (Figure 2D) following primary transmission into RIII mice. UK-2 also showed 2 PrP^Sc^ patterns, the first (Figure 3D) consistent with UK-1 Pattern 1 in RIII mice (Figure 2B) and the second (Figure 3E) consistent with the ME7-like pattern previously described (Figure 3C).

**Figure 4:**
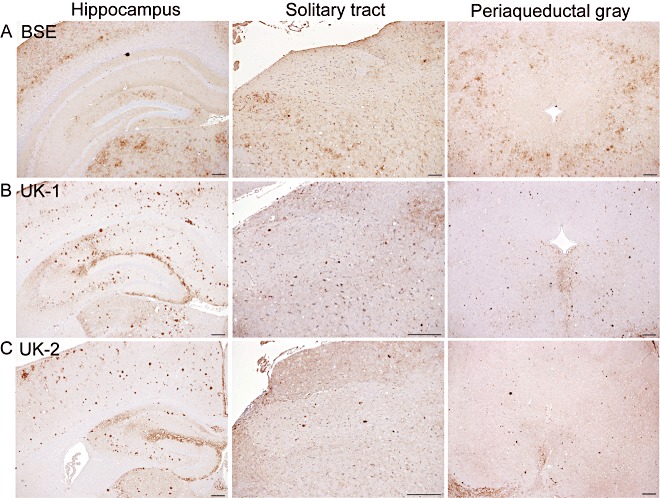
PrP^Sc^ deposition patterns of UK-1 and UK-2 following transmission to VM mice. Representative photographs of PrP^Sc^ deposition in the hippocampus, solitary tract and periaqueductal gray are shown for bovine spongiform encephalopathy (BSE) (**A**), UK-1 (**B**) and UK-2 (**C**). Scale bars represent 100 µm.

The results of IHC analysis following primary isolation in VM mice are shown in Figure 4. BSE (Figure 4A) was characterized by deposition in the hippocampus targeted to the molecular layer of the dentate gyrus. The solitary tract and periaqueductal gray were predominantly spared of PrP^Sc^. UK-1 and UK-2 were clearly distinguishable from BSE and showed a similar pattern to each other (Figure 4B,C). Plaques and aggregates were observed throughout the hippocampus, and deposition was targeted most intensely to the hippocampal fissure. Deposition was observed throughout the solitary tract and the periaqueductal gray. This pattern was designated 87V-like to denote its similarity with the pattern observed with the 87V strain (Supporting Information Figure S4).

IHC analysis following primary isolation in MR mice revealed two predominant PrP^Sc^ patterns, one of which was ME7-like in *Prnp^a^* mice and another pattern that was 87A-like (results not shown). Following transmission of UK-1, half the mice showed an 87A-like PrP^Sc^ pattern while the rest showed an ME7-like pattern. Conversely, the majority of mice inoculated with UK-2 demonstrated the ME7-like pattern. Remaining mice showed a mild 87A-like pattern.

### Western blot analysis following primary isolation in wild-type mice

**Figure 5:**
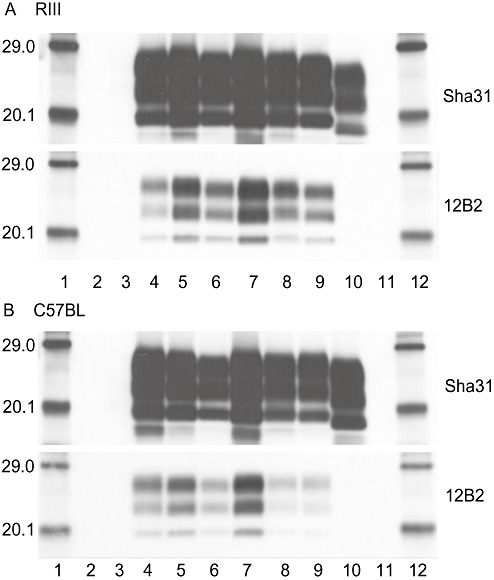
Western blot analysis in wild-type mice. Western blot analysis of proteinase-K-digested PrP^Sc^ from UK-1 and UK-2 challenged RIII (**A**) and C57BL (**B**) mice, detected with Sha31 and 12B2 antibodies. Lanes 1 and 12: molecular mass markers (kDa), Lanes 2 and 11: blank, lane 3: unchallenged proteinase-K digested mouse brain homogenate, lane 4: mouse challenged with ovine classical scrapie, lanes 5–6 and 8–9: mice challenged with UK-1 or UK-2, lane 7: mouse challenged with murine passaged ME7 strain, lane 10: mouse challenged with sheep bovine spongiform encephalopathy (BSE). Exposure time was1 minute for all blots.

Brain material from RIII (Figure 5A) and C57BL (Figure 5B) mice challenged with UK-1 or UK-2 and already diagnosed as positive based on histopathology underwent Western blot analysis. PrP^Sc^ was detected using Sha31 and 12B2 monoclonal antibodies. RIII and C57BL mice inoculated with a natural case of classical ovine scrapie, mouse passaged ME7 and ovine BSE were included as controls. UK-1 and UK-2 samples were selected to represent animals that exhibited each of the different PrP^Sc^ patterns as shown in Figures 2 and 3. Results showed that all UK-1 and UK-2 samples showed an unglycosylated band and reactivity with 12B2 antibody that was consistent with classical scrapie or mouse passaged ME7 and distinct from BSE. We did not subject any VM mice to Western blot analysis as in this line, Western blots can be uninformative in discriminating BSE from scrapie (27, 34).

### Second passage in wild-type mice

**Figure 6:**
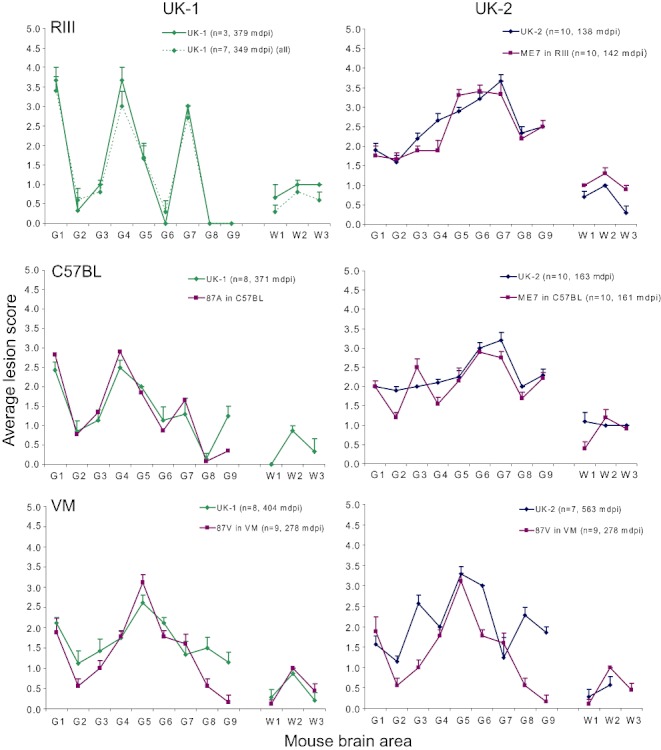
LP following sub-passage of UK-1 and UK-2 in wild-type mice. Lesion profiles (LP) of UK-1 and UK-2 at second passage in RIII, C57BL and VM mouse lines are shown plotted alongside reference classical scrapie strains. Where less than five clinically and histopathologically positive mice were available, separate profiles, representing all haematoxylin and eosin (H&E)-positive mice (dashed line) and mice that were both clinically and H&E positive (solid line) are plotted. Error bars indicate standard error of the mean. The numbers of animals that contributed to each profile and the IP (mean days post inoculation) for each profile are given in parentheses. The 87A in C57BL profile was reproduced from Fraser and Dickinson (23).

IP data and LP following subpassage of UK-1 and UK-2 in wild-type mice are presented in Figure 6. The IP and LP of UK-1 in RIII and C57BL mouse lines were consistent with scrapie strain 87A (23). Analysis of IHC performed on histopathologically positive mice showed that PrP^Sc^ patterns were representative of the donor mouse used for subpassage (results not shown). An exception was noted for subpassage of UK-1 in C57BL mice where the donor mouse showed an 87A-like PrP^Sc^ pattern but the recipient mice showed either an 87A pattern, an ME7 pattern or a possible mixture of the two.

On second passage of UK-2 in RIII and C57BL lines, IP and LP data were consistent with ME7 isolated in the respective mouse lines. IHC analysis of histopathologically positive mice was also consistent with the presence of ME7 (data not shown) and was representative of the donor mouse used for subpassage.

The LP following subpassage of UK-1 and UK-2 in VM mice resembled 87V. This was particularly pertinent for UK-1. However, the UK-2 profile was, in our experience, also within the normal range of variability of 87V. We have previously observed isolates that on second passage, produced similar profiles to UK-2, which on third serial passage, presented a LP compatible with 87V (unpub. obs.). The 87V profile to which UK-1 and UK-2 were compared with in the present study represents 87V after multiple serial passages in VM mice after which its properties are said to “stabilize”(11); indeed, the longer IP observed for UK-1 and UK-2 suggest that full strain stabilization was not yet complete for these sources. IHC analysis of VM mice following second passage of UK-1 and UK-2 produced PrP^Sc^ patterns that were consistent with 87V (data not shown).

### Primary isolation in ovine transgenic mice

The IP of UK-1 and UK-2 following transmission to tg338 mice was not consistent with the IP of ovine BSE in this mouse line, as the latter typically exceeds 500 days on primary passage (Table 2) (8, 9). The mean IP of UK-1 and UK-2 in TgshpXI mice varied from 166 to 196 days while the IP of ovine BSE in this mouse line exceeds 300 days (28). Taken together, these results indicate that UK-1 and UK-2 have biological properties that are inconsistent with ovine BSE. Results of identical bioassays carried out at INRA and FLI were in agreement with the VLA data (Table 2).

**Table 2:** Attack rates and incubations periods of UK-1 and UK-2 following transmission to ovine transgenic mice.

Case ID	Mouse Line	VLA		INRA		FLI	
				
		Nc/N	IP (SD)	Nc/N	IP (SD)	Nc/N	IP (SD)
UK-1	tg338 (all)	16/20	121 (21.9)	12/12	141 (26.2)		
	tg338 (P_338_)*	3/20	158 (5.1)	6/12	161 (10.0)		
	tg333 (G_338_)*	10/20	107 (8.1)	5/12	113 (12.2)		
	tg338 (Composite)*	3/20	130 (1.7)	1/12	155		
	TgshpXI	20/20	184 (29.2)			15/15	166 (19.4)
UK-2	tg338	20/20	149 (3.1)	12/12	143 (5.4)		
	TgshpXI	17/20	164 (7.3)			14/15	196 (67.3)
Classical scrapie	tg338	10/10	154 (3.3)				

* See Figure 8.

Attack rates and mean incubation period (IP) data for transmissions of UK-1 and UK-2 to tg338 and TgshpXI mice carried out at Veterinary Laboratories Agency (VLA), French National Institute for Agricultural Research (INRA) and Friedrich-Loeffler-Institut (FLI) are shown. Classical scrapie data following transmission to tg338 mice is included for comparison. In the case of UK-1, mean IP correlating to the distinct PrP^Sc^ immunohistochemical (IHC) deposition patterns shown by individual mice are also reported (P_338_, G_338_ and composite). Nc indicates the number of mice diagnosed as transmissible spongiform encephalopathy (TSE)-positive that also exhibited clinical signs of TSE; N indicates the number of mice inoculated. SD indicates standard deviation.

**Figure 7:**
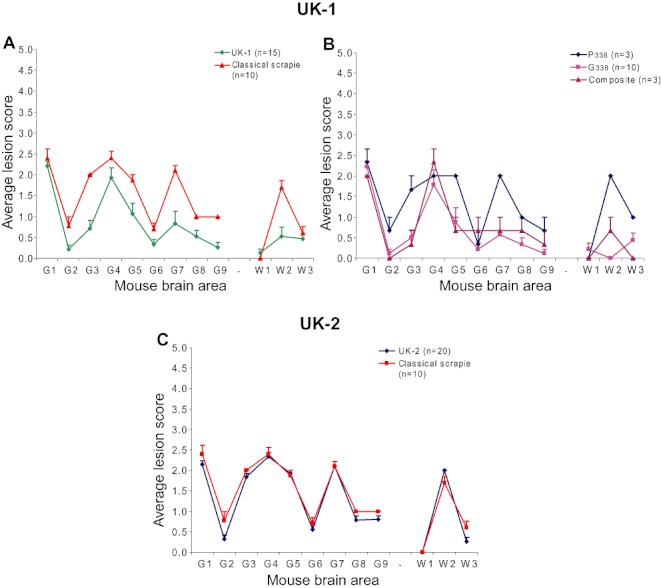
UK-1 and UK-2 LP following primary isolation in ovine transgenic mice. Lesion profiles (LP) following transmission of UK-1 and UK-2 to tg338 mice are shown in (**A**) and (**C**), respectively. Profiles represent mice that were both clinically and haematoxylin and eosin (H&E) positive plotted alongside a classical scrapie LP. Error bars indicate standard error of the mean. In (**B**), UK-1 profiles were re-plotted according to the PrP^Sc^ deposition pattern, denoted P_338_, G_338_ or composite, shown by individual mice.

The average LP given by UK-1 following transmission to tg338 mice, was largely similar to that of an ARQ/ARQ classical scrapie field isolate (Figure 7A) while the LP of UK-2 following transmission to tg338 mice was entirely consistent with this source (Figure 7C).

**Figure 8:**
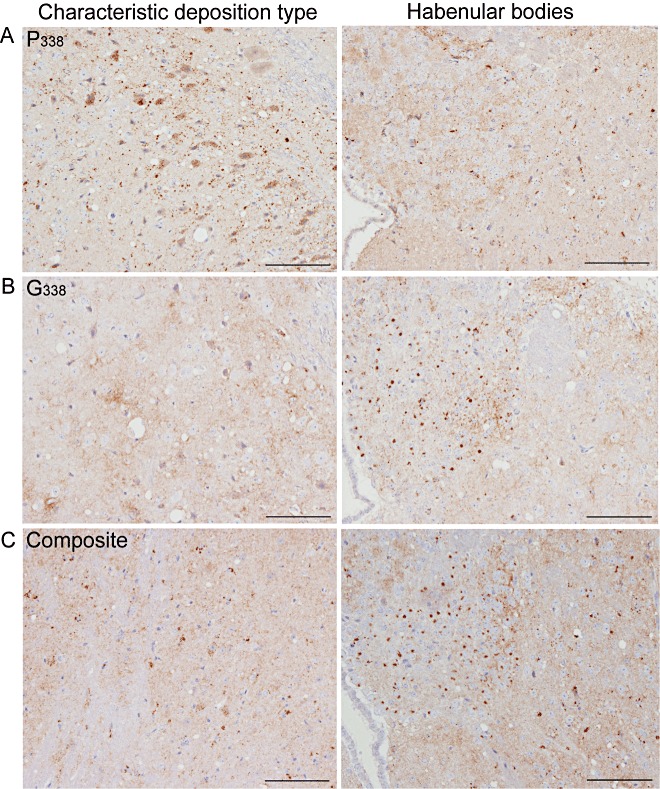
PrP^Sc^ deposition patterns of UK-1 following transmission to tg338 mice. PrP^Sc^ labeling in tg338 mice showed distinct patterns and deposition types. The characteristic deposition type and labeling within the habenular bodies are also shown. P_338_ (**A**) was predominantly punctuate and intraneuronal labeling. G_338_ (**B**) was characterized by granular labeling within the neuropil and distinctive aggregates within the medial habenular bodies. A number of mice showed a mixture of P_338_ and G_338_ (composite) (**C**). Scales bars represent 100 µm.

IHC was performed on H&E-positive tg338 and TgshpXI mice infected with UK-1 and UK-2 isolates. In tg338 mice challenged with UK-1 or UK-2, we observed two distinct patterns of PrP^Sc^ deposition. The first pattern (P_338_) was characterized by intraneuronal, intraglial and punctate labeling in the neuropil located predominantly throughout the medulla, cerebellar nuclei, midbrain, hypothalamic nuclei, thalamus, zona incerta, habenular bodies, septal nuclei and the nucleus of the diagonal band (Figure 8A). The second pattern (G_338_) was distinct from the first and was characterized predominantly by fine granular deposition throughout the neuropil of the medulla, cerebellar nuclei, midbrain (ventrolateral nuclei, raphe and periaqueductal gray), hypothalamic nuclei, thalamus (dorsomedial, including habenular bodies), amygdala, septal nuclei and the nucleus of the diagonal band. Intraneuronal labeling was not observed consistently. When present, it affected large neurons mainly in the medulla and was less distinct compared with the intraneuronal labeling observed in P_338_. Additionally G_338_ pattern was associated with distinctive aggregates of PrP^Sc^ in the medial habenular bodies (Figure 8B). Both P_338_ and G_338_ deposition patterns were previously identified following transmission of classical scrapie isolates in tg338 mice (39, 40).

The majority of mice (n = 10) from UK-1 demonstrated pattern G_338_, while in the rest, we detected either pattern P_338_ (n = 3) or a composite pattern showing features of both P_338_ and G_338_ (n = 3) (Figure 8C). All mice infected with the UK-2 isolate gave a PrP^Sc^ deposition pattern consistent with P_338_. The IP and LP of UK-1 infected tg338 mice were recalculated according to PrP^Sc^ deposition pattern (P_338_, G_338_ or composite) (Figure 7B). IP grouped according to PrP^Sc^ deposition pattern. P_338_ was associated with the longest and G_338_ with the shortest IP. The composite pattern was associated with an intermediate IP between that of P_338_ and G_338_ for those mice inoculated at VLA (Table 2). The profile of P_338_ was consistent with classical scrapie, while G_338_ and the composite patterns were comparable with each other and with the profile derived from all mice shown in Figure 7A, likely owing to the larger sample number contributing to G_338_. All mice inoculated with UK-2 showed P_338_ and had a longer IP consistent with that pattern (Table 2).

A single IHC pattern was observed for both isolates inoculated into TgshpXI mice characterized by widespread intraneuronal, punctate and intraglial labeling (results not shown).

### Western blot analysis following primary isolation in ovine transgenic mice

**Figure 9:**
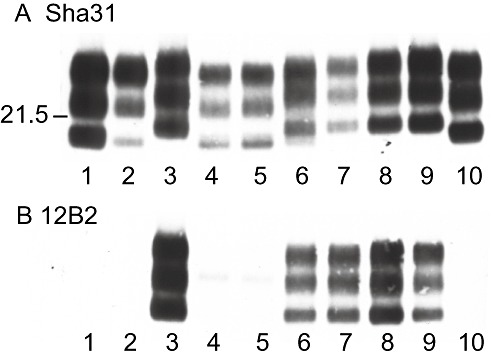
Western blot analysis of ovine transgenic mice inoculated with UK-1. Detection of PrP^Sc^ in proteinase-K-treated brain homogenates by Sha31 and 12B2 monoclonal antibodies. Lane 1: original UK-1 inoculum, lane 2: sheep bovine spongiform encephalopathy (BSE), lane 3: classical scrapie, lanes 4–8: UK-1 inoculated tg338 mouse brains exhibiting (with Sha31 mAb) a 19-kDa profile as referred to the migration of the unglycosylated band (lanes 4–5), a mixed profile (lane 6) or a 21-kDa profile (lanes 7–8), lane 9: classical scrapie in tg338 mice, lane 10: sheep BSE in tg338 mice.

After inoculation with UK-1, Western blot analysis revealed differences in the molecular mass of the lower, unglycosylated PrP^Sc^ band detected by mAb Sha31 among tg338 mice. Figure 9A represents samples resulting from transmissions carried out at INRA, including mouse brains exhibiting a 19-kDa profile [with respect to the migration of the unglycosylated band] (lanes 4 and 5), a mixed profile at approximately 20 kDa (lane 6) or a 21-kDa profile (lanes 7 and 8). Those exhibiting the 19-kDa profile were essentially negative for 12B2 reactivity, consistent with the original ovine inoculum (lane 1) and with BSE in sheep (lane 2) or tg338 mice (lane 10) (Figure 9B). Conversely the 12B2 reactivity of mice showing the 21 kDa or the mixed 20-kDa profile was consistent with classical scrapie in sheep (lane 3) and tg338 mice (lane 9). Results were repeatable at VLA. Samples showing the 19 kDa Western blot profile were found to exhibit the P_338_ PrP^Sc^ pattern previously described (Figure 8). The 21-kDa samples showed the G_338_ pattern while the samples showing a mixed profile on Western blot exhibited a composite PrP^Sc^ pattern of P_338_ and G_338_ also.

All tg338 mice challenged with UK-2 exhibited a 19-kDa unglycosylated band with Sha31 and an absent or much reduced signal with P4, consistent with the original ovine inoculum (Supporting Information Figure S5). Following inoculation with either UK-1 or UK-2, TgShpXI mice gave a 19-kDa unglycosylated band with Sha31 (VLA: Supporting Information Figure S5) or L42 (FLI).

### Sequencing of UK-1 and UK-2 PRNP open reading frame

The full open reading frame of UK-1 and UK-2 was sequenced in order to investigate the presence of additional polymorphisms outside of codons 136, 154 and 171 that might be attributed to the ambiguous nature of these samples. The UK-1 sequence showed an additional heterozygous non-synonymous polymorphism at codon 241 (P241S). This polymorphism has been previously associated with the ARQ allele (35). The UK-2 sequence showed an additional homozygous non-synonymous polymorphism at codon 141 (L141F). Polymorphisms at codon 141 are associated with susceptibility to Nor98 scrapie and are rare in classical scrapie cases (7, 35).

## DISCUSSION

The aim of this study was to determine the properties of the agent(s) present in two cases of TSE in sheep, UK-1 and UK-2, which did not conform to the definition of classical scrapie or Nor98 and shared some similarities with BSE (37). The results of a comprehensive mouse bioassay utilizing wild-type and ovine transgenic mice confirmed these cases to be compatible with classical scrapie rather than with BSE in sheep. The presence of the BSE agent was contraindicated by IP in transgenic mice, LP on primary isolation in RIII and/or MR mice, LP and IP on subpassage in wild-type mice and PrP^Sc^ deposition pattern in wild-type mice. In addition, the investigation of UK-1 and UK-2 also provided a platform on which to demonstrate the merits of utilizing the mouse bioassay to investigate unusual TSE cases and an opportunity to compare the choice of mouse lines that such investigations might employ.

In the past, LP during primary isolation in RIII mice have been used to discriminate BSE from classical scrapie and establish a link between BSE and vCJD (15, 16, 26). However, it has recently been reported that ovine sources, derived from ARQ/ARQ sheep, can produce LP, termed 1-4-7-scrapie, which cannot be distinguished from BSE based on this parameter (3). UK-1 LP also presented with a “1-4-7” pattern on primary isolation in RIII mice that was indistinguishable from BSE using standard LP methodology (26). Here, we showed that by modifying the standard protocol, the LP were distinguishable. PrP^Sc^ distribution patterns and Western blot data can be used to differentiate this 1-4-7-scrapie from BSE. In addition to the three traditional wild-type mouse lines used for TSE strain typing two other lines, MR and SJL/OlaHsd were also assessed. MR mice challenged with UK-1 or UK-2 generated LP that were clearly distinguishable from the characteristics of BSE and similar to the ME7 LP. Conversely SJL/OlaHsd was not a useful model for these studies; UK-1 did not transmit, while the UK-2 LP was indistinguishable from BSE, albeit with an extended IP compared with BSE (data not shown).

IP, LP and IHC analysis of primary and serial passages of UK-1 and UK-2 in wild-type mice revealed isolation of ME7 and 87A strains in RIII and C57BL mouse lines and 87V in VM mice. The PrP^Sc^ patterns associated with these strains were also recognizable as early as primary isolation on an individual mouse basis. This supports a recent study in which we reported the presence of an ME7-like agent on primary isolation in wild-type mice (4). Western blots of RIII and C57BL mice were characteristic of classical scrapie, and the profiles of mice exhibiting ME7-like and 87A-like PrP^Sc^ patterns were indistinguishable by this method.

The data emerging from the bioassays in the transgenic mice demonstrate isolation of two agents, one of which had a 19-kDa Western blot profile typical of BSE and consistent with the Western blot profile of the original ovine inoculum. However, the LP and IP were consistent with classical scrapie. The other had the biochemical and biological properties of classical scrapie. The fact that transgenic mice with the 19-kDa Western blot profile showed biological properties of scrapie meant that the possibility that a BSE agent had coexisted but remained undetected could be rejected. Similar cases of classical scrapie with a 19-kDa unglycosylated band have been reported in France (41). The 19-kDa and 21-kDa Western blot profiles in tg338 mice were associated with specific PrP^Sc^ patterns, P_338_ and G_338_ that were also observed in a previous study (39), where the same patterns and also stabilised IP continued to be observed on further passages (40). Taken together, these results suggest the presence of two scrapie strains. Previous studies have shown that more that one prion strain can be isolated from homogenous ovine samples passaged in wild-type and transgenic mice (32, 40, 47, 48).

In wild-type mice, while the same two PrP^Sc^ patterns, 87A-like and ME7-like, were observed in RIII and C57BL mice on primary isolation, the number of mice presenting each pattern was variable between UK-1 and UK-2 in each mouse line. Similar observations have been reported previously (4). An 87A-like PrP^Sc^ pattern appeared to dominate over the ME7-like pattern in RIII mice inoculated with UK-1. This agent would account for the “1-4-7” LP observed in RIII mice. Interestingly the proportion of MR mice showing the ME7-like PrP^Sc^ pattern was greater than that observed in RIII mice. Consequently the LP of UK-1 in MR mice shares similarities with ME7. For UK-2 the majority of RIII and MR mice showed an ME7-like PrP^Sc^ pattern resulting in an ME7-like LP in both RIII and MR mice. The variation in propensity for mice to present with each pattern may be related to the relative titer of each of these strains in the ovine host in combination with their speed of propagation in the mice. According to the conformational hypothesis, it has been suggested that the ease of propagation into mice depends on the ability of the donor prion to induce a conformational change in a given host prion (1). This process is likely to be governed by the conformational flexibility of the host prion protein [reviewed (10)] which may in turn be dependent on mouse line.

Facilitated transmission of UK-1 and UK-2 in transgenic mice highlighted the differential sensitivity of these models in detecting possible mixtures of strains. The tg338 line, which carries the VRQ transgene, detected two strains in UK-1 in accordance with wild-type mice but only one strain in UK-2. Conversely, TgshpXI mice were only able to detect a single strain for each isolate. A possible explanation of these results could be that there were two strains in the original host that were successfully isolated in RIII, C57BL, MR and tg338 mice while in VM and TgshpXI mice, only one of the pre-existing strains was propagated (12). Alternatively if a TSE agent is propagated on a non-homologous PrP^C^ substrate, it may adopt more than one conformation, giving rise to different strains. According to this hypothesis, the isolation of a single strain from UK-1 and UK-2, which both represent ARQ/ARQ sources in the TgshpXI mouse line that carries the ARQ transgene, was expected. However, more than one strain can emerge after transmission of classical scrapie sources to homologous PrP^C^ transgenic mouse lines (own unpub. obs.).

This study highlighted that during primary isolation, more than one strain may be isolated from a single source, and for this reason, identification of strains on an individual animal basis during primary isolation may better reflect the true repertoire of agents in the original host. In this respect, IP and LP may be less reliable parameters for strain identification as they represent mean values of a group of observations. Also in serial passages, a single animal is used as a donor, and generally, the phenotype of this animal is propagated, resulting in selection of the dominant strain in the donor animal.

Our data also suggest that strain competition is not absolute where one strain prevails over another as following transmission of a single source into a panel of mice, one group of mice may propagate one strain, another group may propagate another strain and yet some mice may present features of both. This was particularly evident in our study where we were able to identify single animals showing both P_338_ and G_338_. We believe that this is contrary to previously published data because in our study, strain identification was based on single animals (19, 20). Our data are in agreement with the findings of a recent study reporting on an ovine scrapie isolate that contained two strains, one of which appeared to mask the presence of the other (47). Subsequent intraspecies (sheep to sheep) as well as interspecies (sheep to mouse) transmissions resulted in selection and isolation of individual strains.

In conclusion, results from the mouse bioassay confirm that UK-1 and UK-2 are consistent with classical scrapie. Since bioassay data for ovine passaged atypical (H- and L- type) BSE are not available at this time, conclusions cannot be made regarding these BSE variants. While BSE has not been identified in sheep to date, further investigation of ambiguous cases remains vital as experimental transmission of BSE to sheep is possible (5, 22). Utilizing a combined approach and analyzing biological and biochemical aspects of transmission, we have not only concluded that UK-1 and UK-2 are scrapie, but we are able to hypothesize that each isolate comprised two strains that most likely coexisted in the natural host based on the wild-type and particularly the transgenic mouse data. In our view, the use of transgenic mouse lines is preferable for such investigations. This is because there is no transmission barrier if appropriate lines have been selected, giving a higher attack rate and a greater assurance that what is isolated in the mouse better reflects the agent in the sheep. While different isolates will undoubtedly present different challenges, further expanding our current knowledge regarding the discrimination of TSE agents in wild-type and transgenic mouse models will permit the continued, effective discrimination of TSE strains via the bioassay, using the most suitable mouse lines to reach an unequivocal diagnosis.

## SUPPORTING INFORMATION

**Figure S1.** Changes to the lesion scoring protocol allows UK-1 to be distinguished from bovine spongiform encephalopathy (BSE). Lesion profiles (LP) following transmission of UK-1, ovine and bovine BSE to RIII mice. The G1 scoring area was split so that the dorsal medulla nuclei (G1d) and the cochlear nuclei (G1c) were scored independently. The numbers of animals that contributed to each profile are given in parentheses. Error bars indicate standard error of the mean. Mice were all clinically and pathologically positive.

**Figure S2.** Retrospective analysis of ten classical scrapie sources using the alternative lesion scoring protocol. Lesion profiles (LP) following transmission of 10 classical scrapie sources (red lines) and 10 bovine spongiform encephalopathy (BSE) sources (black lines) to RIII mice as previously detailed (3). The dorsal medulla nuclei (G1d) and the cochlear nuclei (G1c) were scored independently. Error bars indicate standard error of the mean. Only mice with both dorsal medulla and cochlear nucleus present were included. Mice were all clinically and pathologically positive. Each profile was constructed from at least five mice.

**Figure S3.** UK-1 and UK-2 LP followingtransmission to MR and SJL/OlaHsd mouse lines. LP followingtransmission of UK-1 (**A**) and UK-2(**B**) to MR mice and UK-2 to SJL/OlaHsd mice(**C**), are shown. Where less than five clinicallyand histopathologically positive mice were available, separateprofiles representing all hematoxylin and eosin (H&E)-positivemice (dashed line) and mice that were both clinically andH&E-positive (solid line) are plottedalongside representativebovine spongiform encephalopathy (BSE) profiles. Error barsindicate standard error of the mean. The numbers of animals whichcontributed to each profile are given in parentheses. UK-1 did nottransmit to SJL/OlaHsd mice.

**Figure S4.** PrP^Sc^ patterns associatedwith scrapie strains 87A, 87V and ME7. **A.** 87A in aC57BL mouse. **B.** 87V in a VM mouse.**C.** ME7 in an RIII mouse. Scale bars represent 500μm.

**Figure S5.** Western blot analysis of UK-2transmissions to tg338 mice and UK-1 and UK-2 transmissions toTgshpXI mice. **A.** Detection of PrP^Sc^ bySha31 and P4 monoclonal antibodies in proteinase-K treated tg338brain homogenates following challenge with UK-2. Lane 1: originalovine inoculum, lanes 2–11: inoculated tg338 mouse brains,lanes 12 and 15: bovine BSE control, lane 13: experimental bovinespongiform encephalopathy (BSE) in sheep control, lane 14:classical scrapie control. **B.** Detection ofPrP^Sc^ by Sha31 and P4 monoclonal antibodies inproteinase-K treated TgShpXI brain homogenates following challengewith UK-1 and UK-2. Lanes 1–4 and 7–10: TgshpXI miceinoculated with UK-1 and UK-2, respectively. Lane 5: UK-1challenged tg338 mouse, lane 6: blank, lane 11: UK-2 challengedtg338 mouse, lanes 12–13: unchallenged TgshpXI mouse−/+ proteinase-K, lane 14: bovine BSE, lane 15: ovinescrapie. Molecular mass markers (in kDa).

## References

[1] Aguzzi A (1998). Protein conformation dictates prion strain. Nat Med.

[2] Asante EA, Linehan JM, Desbruslais M, Joiner S, Gowland I, Wood AL (2002). BSE prions propagate as either variant CJD-like or sporadic CJD-like prion strains in transgenic mice expressing human prion protein. EMBO J.

[3] Beck KE, Chaplin M, Stack M, Sallis RE, Simonini S, Lockey R (2010). Lesion profiling at primary isolation in RIII mice is insufficient in distinguishing BSE from classical scrapie. Brain Pathol.

[4] Beck KE, Sallis RE, Lockey R, Simmons MM, Spiropoulos J (2010). Ovine PrP genotype is linked with lesion profile and immunohistochemistry patterns after primary transmission of classical scrapie to wild-type mice. J Neuropathol Exp Neurol.

[5] Bellworthy SJ, Dexter G, Stack M, Chaplin M, Hawkins SAC, Simmons MM (2005). Natural transmission of BSE between sheep within an experimental flock. Vet Rec.

[6] Bellworthy SJ, Dexter G, Stack M, Chaplin M, Hawkins SAC, Simmons MM (2008). Oral transmission of BSE to VRQ/VRQ sheep in an experimental flock. Vet Rec.

[7] Benestad SL, Arsac JN, Goldmann W, Noremark M (2008). Atypical/Nor98 scrapie: properties of the agent, genetics, and epidemiology. Vet Res.

[8] Beringue V, Bencsik A, Le DA, Reine F, Lai TL, Chenais N (2006). Isolation from cattle of a prion strain distinct from that causing bovine spongiform encephalopathy. PLoS Pathog.

[9] Beringue V, Andreoletti O, Le DA, Essalmani R, Vilotte JL, Lacroux C (2007). A bovine prion acquires an epidemic bovine spongiform encephalopathy strain-like phenotype on interspecies transmission. J Neurosci.

[10] Beringue V, Vilotte JL, Laude H (2008). Prion agent diversity and species barrier. Vet Res.

[11] Bruce ME (1993). Scrapie strain variation and mutation. Br Med Bull.

[12] Bruce ME (2003). TSE strain variation: an investigation into prion disease diversity. Br Med Bull.

[13] Bruce ME, McConnell I, Fraser H, Dickinson AG (1991). The disease characteristics of different strains of scrapie in Sinc congenic mouse lines: implications for the nature of the agent and host control of pathogenesis. J Gen Virol.

[14] Bruce M, Chree A, McConnell I, Foster J, Pearson G, Fraser H (1994). Transmission of bovine spongiform encephalopathy and scrapie to mice: strain variation and the species barrier. Philos Trans R Soc Lond B Biol Sci.

[15] Bruce ME, Will RG, Ironside JW, McConnell I, Drummond D, Suttie A (1997). Transmissions to mice indicate that “new variant” CJD is caused by the BSE agent. Nature.

[16] Bruce ME, Boyle A, Cousens S, McConnell I, Foster J, Goldmann W (2002). Strain characterization of natural sheep scrapie and comparison with BSE. J Gen Virol.

[17] Community RL (2005). http://vla.defra.gov.uk/science/docs/sci_tse_rl_prp_ihc.pdf.

[18] Cunningham AA, Kirkwood JK, Dawson M, Spencer YI, Green RB, Wells GA (2004). Bovine spongiform encephalopathy infectivity in greater kudu (*Tragelaphus strepsiceros*. Emerg Infect Dis.

[19] Dickinson AG, Fraser H, Meikle VM, Outram GW (1972). Competition between different scrapie agents in mice. Nat New Biol.

[20] Dickinson AG, Fraser H, McConnell I, Outram GW, Sales DI, Taylor DM (1975). Extraneural competition between different scrapie agents leading to loss of infectivity. Nature.

[21] European Commission (2005). http://eur-lex.europa.eu/LexUriServ/LexUriServ.do?uri=OJ:L:2005:010:0009:0017:EN:PDF.

[22] Foster JD, Hope J, Fraser H (1993). Transmission of bovine spongiform encephalopathy to sheep and goats. Vet Rec.

[23] Fraser H, Dickinson AG (1973). Scrapie in mice. Agent-strain differences in the distribution and intensity of grey matter vacuolation. J Comp Pathol.

[24] Gavier-Widén D, Nöremark M, Langeveld JP, Stack M, Biacabe AG, Vulin J (2008). Bovine spongiform encephalopathy in Sweden: an H-type variant. J Vet Diagn Invest.

[25] Gonzalez L, Chianini F, Martin S, Siso S, Gibbard L, Reid HW (2007). Comparative titration of experimental ovine BSE infectivity in sheep and mice. J Gen Virol.

[26] Green R, Horrocks C, Wilkinson A, Hawkins SA, Ryder SJ (2005). Primary isolation of the bovine spongiform encephalopathy agent in mice: agent definition based on a review of 150 transmissions. J Comp Pathol.

[27] Groschup MH, Kuczius T, Junghans F, Sweeney T, Bodemer W, Buschmann A (2000). Characterization of BSE and scrapie strains/isolates. Arch Virol Suppl.

[28] Kupfer L, Eiden M, Buschmann A, Groschup MH (2007). Amino acid sequence and prion strain specific effects on the *in vitro* and *in vivo* convertibility of ovine/murine and bovine/murine prion protein chimeras. Biochim Biophys Acta.

[29] Laude H, Vilette D, Le DA, Archer F, Soulier S, Besnard N (2002). New *in vivo* and *ex vivo* models for the experimental study of sheep scrapie: development and perspectives. C R Biol.

[30] Le Dur A, Beringue V, Andreoletti O, Reine F, Lai TL, Baron T (2005). A newly identified type of scrapie agent can naturally infect sheep with resistant PrP genotypes. Proc Natl Acad Sci U S A.

[31] Pan KM, Baldwin M, Nguyen J, Gasset M, Serban A, Groth D (1993). Conversion of alpha-helices into beta-sheets features in the formation of the scrapie prion proteins. Proc Natl Acad Sci U S A.

[32] Polymenidou M, Stoeck K, Glatzel M, Vey M, Bellon A, Aguzzi A (2005). Coexistence of multiple PrPSc types in individuals with Creutzfeldt-Jakob disease. Lancet Neurol.

[33] Prusiner SB (1991). Molecular biology of prion diseases. Science.

[34] Ritchie DL, Boyle A, McConnell I, Head MW, Ironside JW, Bruce ME (2009). Transmissions of variant Creutzfeldt-Jakob disease from brain and lymphoreticular tissue show uniform and conserved bovine spongiform encephalopathy-related phenotypic properties on primary and secondary passage in wild-type mice. J Gen Virol.

[35] Saunders GC, Cawthraw S, Mountjoy SJ, Hope J, Windl O (2006). PrP genotypes of atypical scrapie cases in Great Britain. J Gen Virol.

[36] Scott MR, Will R, Ironside J, Nguyen HO, Tremblay P, DeArmond SJ (1999). Compelling transgenetic evidence for transmission of bovine spongiform encephalopathy prions to humans. Proc Natl Acad Sci U S A.

[37] Stack M, Jeffrey M, Gubbins S, Grimmer S, Gonzalez L, Martin S (2006). Monitoring for bovine spongiform encephalopathy in sheep in Great Britain, 1998–2004. J Gen Virol.

[38] Stack M, Jeffrey M, Deslys J-P, Grassi J, Baron T, Safar J (2008). http://vla.defra.gov.uk/science/docs/sci_tse_rl_ringtrial.pdf.

[39] Thackray AM, Hopkins L, Spiropoulos J, Bujdoso R (2008). Molecular and transmission characteristics of primary-passaged ovine scrapie isolates in conventional and ovine PrP transgenic mice. J Virol.

[40] Thackray AM, Hopkins L, Lockey R, Spiropoulos J, Bujdoso R (2011). Emergence of multiple prion strains from single isolates of ovine scrapie. J Gen Virol.

[41] Tixador P, Herzog L, Reine F, Jaumain E, Chapuis J, Le Dur A (2010). The physical relationship between infectivity and prion protein aggregates is strain-dependent. PLoS Pathog.

[42] Westaway D, Goodman PA, Mirenda CA, McKinley MP, Carlson GA, Prusiner SB (1987). Distinct prion proteins in short and long scrapie incubation period mice. Cell.

[43] Wilesmith JW, Wells GA, Cranwell MP, Ryan JB (1988). Bovine spongiform encephalopathy: epidemiological studies. Vet Rec.

[44] Wilesmith JW, Ryan JB, Atkinson MJ (1991). Bovine spongiform encephalopathy: epidemiological studies on the origin. Vet Rec.

[45] World Organisation For Animal Health (2007). http://vla.defra.gov.uk/science/docs/sci_tse_rl_handbookv4jan10.pdf.

[46] Wyatt JM, Pearson GR, Smerdon TN, Gruffydd-Jones TJ, Wells GA, Wilesmith JW (1991). Naturally occurring scrapie-like spongiform encephalopathy in five domestic cats. Vet Rec.

[47] Yokoyama T, Masujin K, Schmerr MJ, Shu Y, Okada H, Iwamaru Y (2010). Intraspecies prion transmission results in selection of sheep scrapie strains. PLoS ONE.

[48] Yull HM, Ritchie DL, Langeveld JP, van Zijderveld FG, Bruce ME, Ironside JW (2006). Detection of type 1 prion protein in variant Creutzfeldt-Jakob disease. Am J Pathol.

