# Supplementary figures and images for: Use of Murine Bioassay to Resolve Ovine Transmissible Spongiform Encephalopathy Cases Showing a Bovine Spongiform Encephalopathy Molecular Profile
Source: Brain Pathol. 2012 May;22(3):265–79. doi: 10.1111/j.1750-3639.2011.00526.x (PMC3505794; doi:10.1111/j.1750-3639.2011.00526.x)

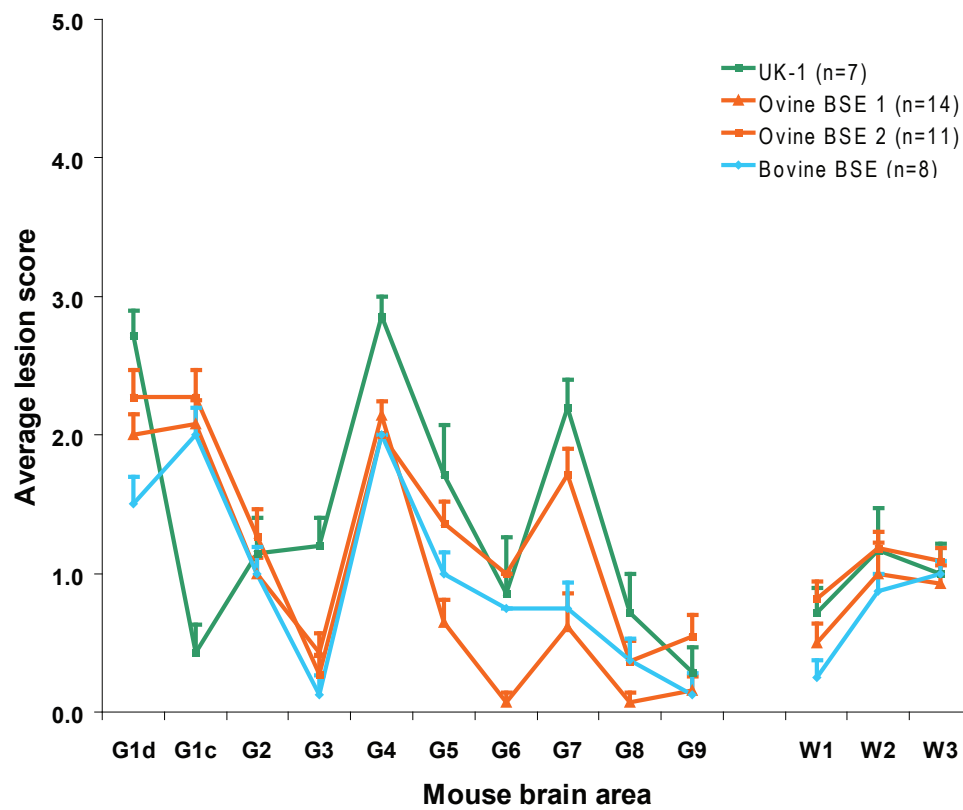

Supplement: Supplementary file 1 [file bpa0022-0265-SD1.pdf]

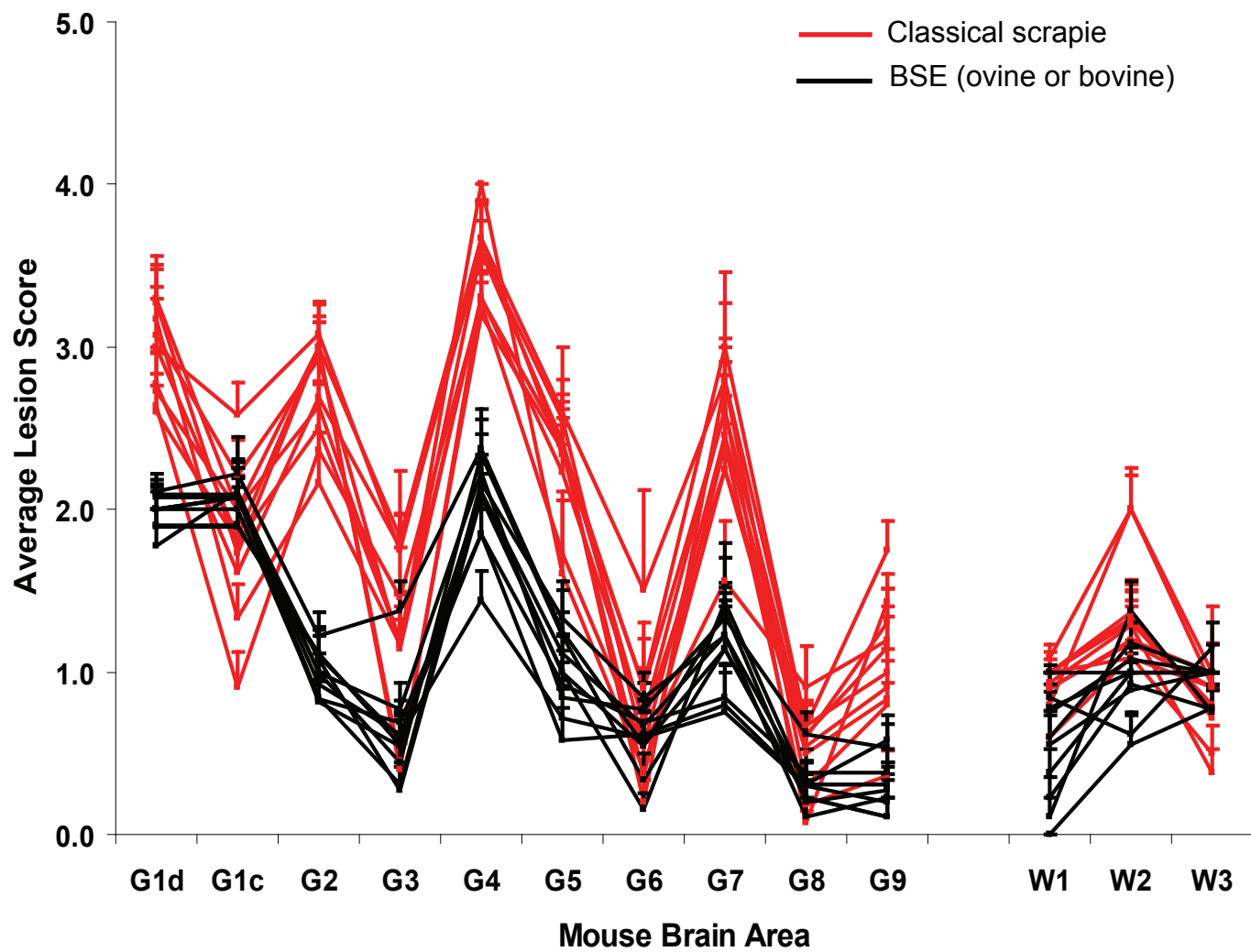

Supplement: Supplementary file 2 [file bpa0022-0265-SD2.pdf]

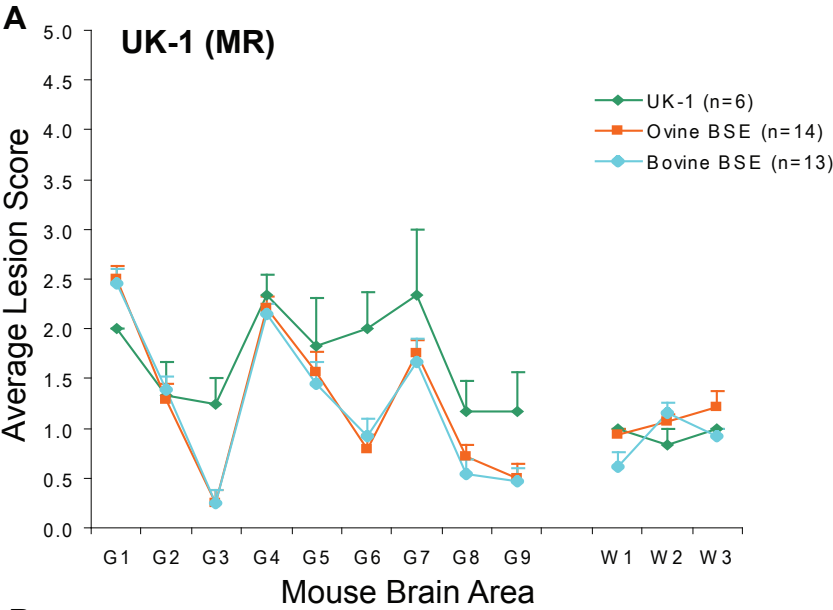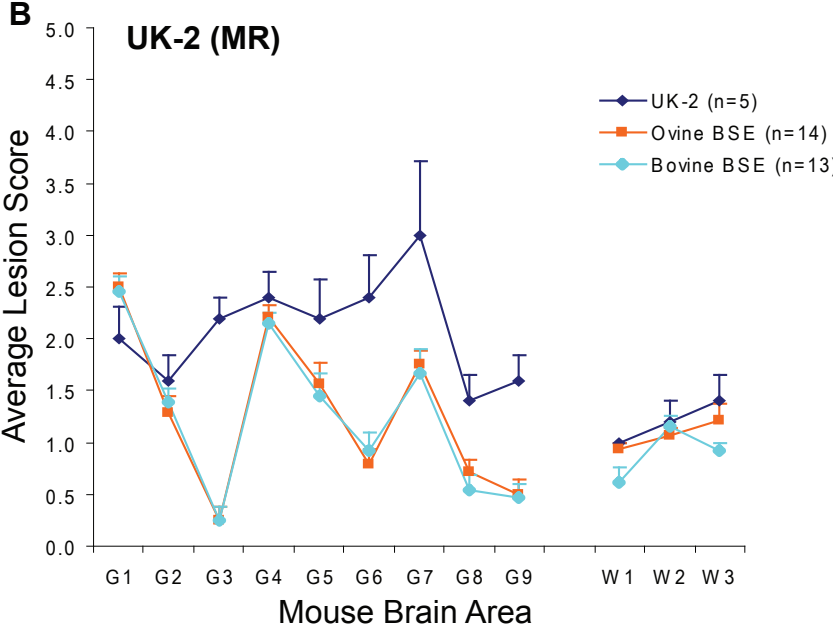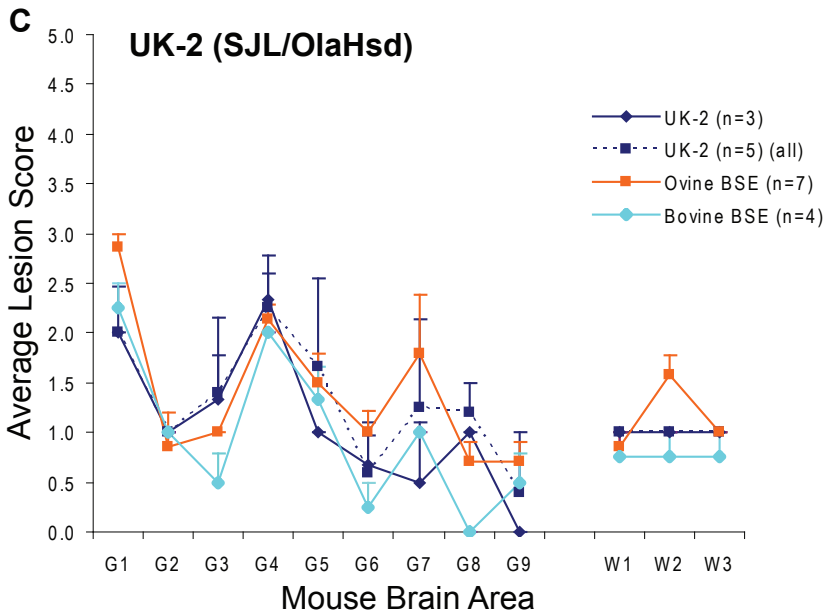

Supplement: Supplementary file 3 [file bpa0022-0265-SD3.pdf]

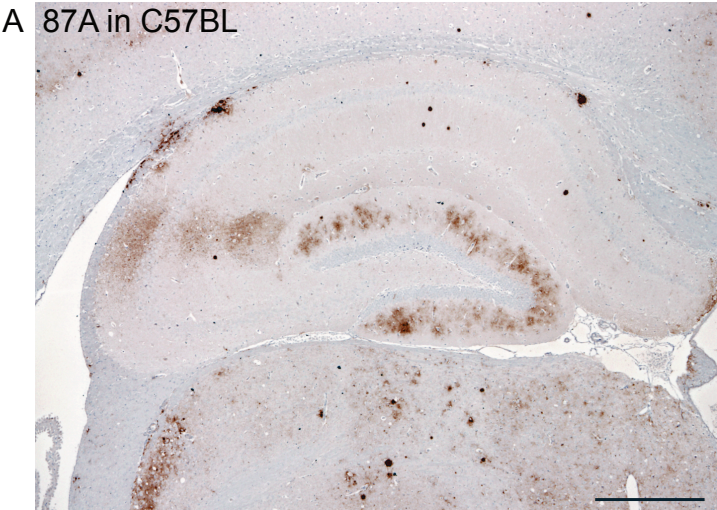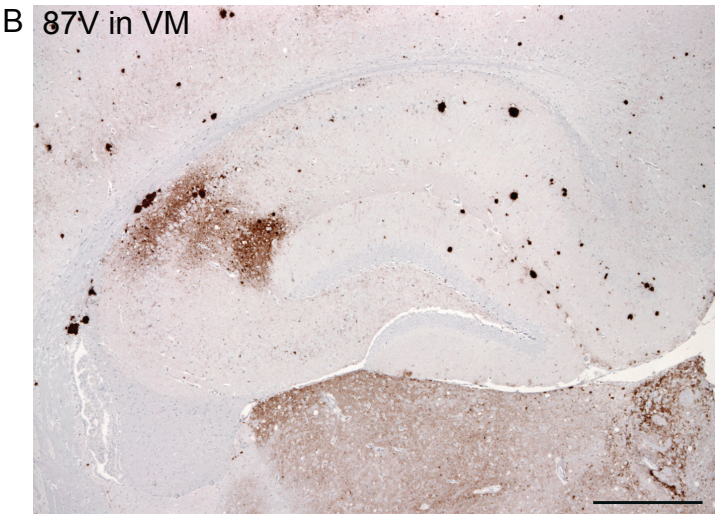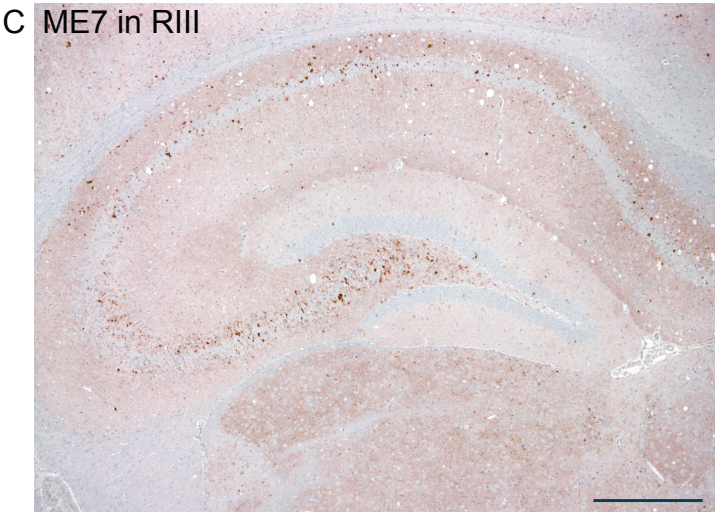

Supplement: Supplementary file 4 [file bpa0022-0265-SD4.pdf]

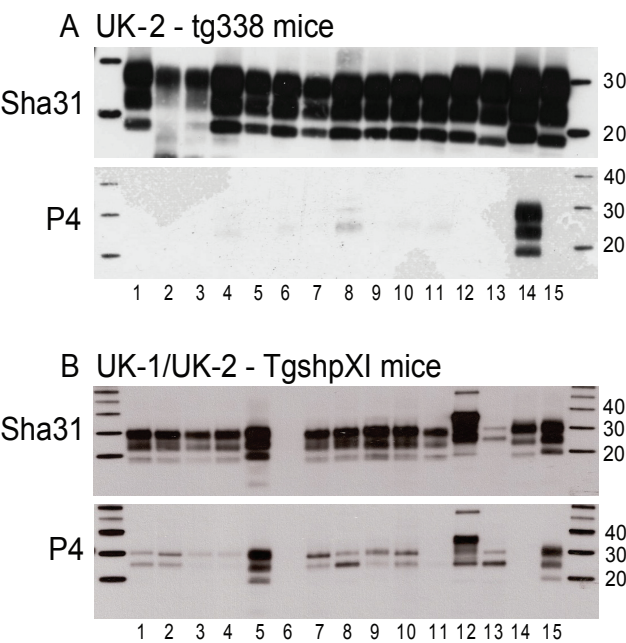

Supplement: Supplementary file 5 [file bpa0022-0265-SD5.pdf]
